# Fraudulent Data Detection and Prevention Within the National Caregiver Survey

**DOI:** 10.1093/geroni/igab046.2935

**Published:** 2021-12-17

**Authors:** Jada Jackson, Jessica Lipchin, Rachel Zhang, Sheria Robinson-Lane

**Affiliations:** 1 University of Michigan School of Nursing, Ann Arbor, Michigan, United States; 2 College of Engineering, University of Michigan Ann Arbor, University of Michigan Ann Arbor, Michigan, United States

## Abstract

The National Caregiver Survey is an online, incentivized survey that aims to gather information about the health and coping strategies used by Black family caregivers of persons with dementia. The survey data will help elucidate the relationships between coping, health, and adaptation to family caregiving and facilitate the development of culturally responsive caregiver support programming. Virtually distributing this survey made it cost-effective, easily accessible, and quick to produce usable data. The online format also helped the team reach caregivers from across the nation, as well as keep participants safe during the COVID-19 pandemic. Unfortunately, because online surveys are advertised and administered digitally, they become targets for hacking, especially when the surveys are incentivized. The hacking attempts are executed through digital survey bots and threaten the integrity of the collected data. These corrupt responses also increase study costs by falsely rewarding the hackers for their survey responses and research team time in the investigation, detection, and removal of fraudulent responses. To detect potential bots, a reCAPTCHA bot system was incorporated into the survey, and survey questions were formatted specifically to thwart hacking attempts. Finally, data were cleaned to remove illogical, inconsistent, and duplicative surveys. Findings from this work may help researchers improve online survey design and data collection methods to provide greater confidence in conclusions drawn from virtually surveyed data.

